# Robot Responsibility and Moral Community

**DOI:** 10.3389/frobt.2021.768092

**Published:** 2021-11-22

**Authors:** Dane Leigh Gogoshin

**Affiliations:** Department of Practical Philosophy, RADAR Research Group, Faculty of Social Sciences, University of Helsinki, Helsinki, Finland

**Keywords:** moral responsibility, artificial moral agency, human-robot interaction, artificial intelligence, accountability structures

## Abstract

It is almost a foregone conclusion that robots cannot be morally responsible agents, both because they lack traditional features of moral agency like consciousness, intentionality, or empathy and because of the apparent senselessness of holding them accountable. Moreover, although some theorists include them in the moral community as moral patients, on the Strawsonian picture of moral community as requiring moral responsibility, robots are typically excluded from membership. By looking closely at our actual moral responsibility practices, however, I determine that the agency reflected and cultivated by them is limited to the kind of moral agency of which some robots are capable, not the philosophically demanding sort behind the traditional view. Hence, moral rule-abiding robots (if feasible) can be sufficiently morally responsible and thus moral community members, despite certain deficits. Alternative accountability structures could address these deficits, which I argue ought to be in place for those existing moral community members who share these deficits.

## 1 Introduction

Since P. F. Strawson’s landmark essay, “Freedom and Resentment” (Strawson, 2008), morally responsible agency is taken to be a matter of being a fitting target of our responsibility practices.^1^ What exactly this fittingness consists in varies by account, but in most basic terms, per Strawson (see also Wallace, 1994), it is an agent’s capacity to fulfill society’s basic normative demands and expectations. This capacity is instantiated in the practices of being held to account when we transgress or exceed, respectively, these demands and expectations. Our actual practices are thus taken as reflections of this capacity – i.e., of responsible agency. On my analysis (see also Gogoshin, 2020), these standards are much lower than those we traditionally associate with human moral agency or the standards which human agents are, in principle, capable of meeting. Rather than requiring robust moral reasons-responsiveness or autonomy, these practices require only sensitivity to them (a sensitivity to the sting of moral disapproval, condemnation, blame and punishment and to the pleasure of moral approval, praise, and reward). In turn, they reflect and cultivate a limited kind of moral agency, one concerned with performance – behavior that conforms to moral values – not with “what’s going on on the inside” (agents’ reasons and intentions).

Should one have the capacity to reliably behave in accordance with the normative demands and expectations of one’s social environment, one is thus morally responsible. On this basis, I argue that autonomous robots^2^ (henceforth just “robots”) who have the capacity to reliably behave in accordance with the relevant moral rules and values of their social environment (henceforth “moral rule-abiding robots”)^3^ are morally responsible agents. As a consequence, on the view that moral community membership is a matter of morally responsible agency (Strawson, 2008; Darwall, 2006)^4^, such robots are moral community members too.^5^ If this result is objectionable, then we ought to add further conditions to moral community membership than morally responsible agency (e.g., sentience^6^). If, however, we retain responsibility as a necessary condition of moral community, by defining it in any more demanding terms than those I lay out in this paper, we would likely have to reject many current members from our moral communities.^7^

This conception of moral agency is clearly in tension with a deeper, more substantive conception of morally responsible agency^8^ – the one at stake in the free will debate – which is rooted in concerns about fairness and desert, for our identity as responsible, rational and in some way free agents (Holroyd, 2007), and for our ultimate moral aspirations. After all, only one who meets certain epistemic and control conditions and/or can meaningfully identify with their actions or attitudes can be blame- or praiseworthy. Furthermore, we value the capacity to recognize and respond to moral reasons. However, this level of agency is neither reflected nor cultivated by our responsibility practices. Accordingly, morally responsible agency falls short of full-blown, autonomous moral agency. I hypothesize that it is obtained, when it is, through a multiplicity of other factors which lie outside of the moral responsibility system. However, in order to meet the basic demands of morality and to function as a moral community (at least in the way that we do), this level of moral agency appears to be unnecessary and, what’s more, given that the other factors behind our moral development are likely non-ubiquitous and contingent (i.e., dependent on one’s environment, upbringing, socioeconomic status, cultural influences, education level, etc.), too demanding.

I proceed as follows. In Section 2, I situate my approach within the existing artificial moral agency debate. In Sections 3 and 4, I present my analysis of the moral responsibility practices, showing that 1) rather than agents’ reasons for action, they reflect agents’ capacity to comply with moral norms, and 2) insofar as they are regulative, they are largely conditioning practices which are limited to regulating behavior. I identify the limitations on moral agency of behavioral regulation. In Section 5, I argue that moral rule-abiding robots can meet the behavioral level of moral agency required for moral community membership and offer some additional reasons to support their membership. In Section 6, I explore potential objections to this argument and offer some solutions. I conclude in Section 7.

## 2 Situating the Proposed View

Although many theorists hold onto the traditional conception of full-blown moral agency as being a matter of moral responsibility (e.g., Sparrow, 2007; Parthemore and Whitby, 2013; Hakli and Mäkelä, 2019), thereby denying robots full-blown moral agency, in the words of Wendell Wallach (Wallach and Allen, 2009), artificial moral agents are necessary and inevitable. Since Floridi and Sanders (2004), there has been a growing trend to divorce the question of moral agency from moral responsibility specifically and from philosophical personhood more generally (see also Sullins, 2006), in order to expand the set of moral agents. This move eliminates distinctly human capacities such as consciousness from the necessary conditions of moral agency.^9^ It is thus generally thought that robots cannot be responsible in the way that mature, neurotypical humans are. Along with recent proposals by Christian List (2021) and Daniel Tigard (2021), which I will address at the end of this section, the present proposal challenges that notion.

The current state of the artificial moral agency debate is laid out in detail in Behdadi and Munthe (2020); I will not attempt to reconstruct it here. As they note, the debate is largely divided into two approaches – the standard or traditional (cf. Johnson, 2006) and the functionalist (cf. Floridi and Sanders, 2004).^10^ The first seeks to identify features of traditional moral agency and to determine whether robots might have them. The second seeks to identify whether the functions of moral agency can be fulfilled by robots. According to Behdadi and Munthe, these two views are rife with conceptual confusion and are hopelessly irreconcilable. They propose shifting the debate away from a determination of whether machines are moral agents and toward which, whether and to what extent they should become part of society.^11^

There are two alternative approaches of particular relevance to my proposed account – those of Mark Coeckelbergh (2009) and John Danaher (2020). They look to see whether robots could be the fitting targets – in some way – of our existing social practices as they relate to moral patiency, agency, or responsibility. They then take facts about those practices, namely that they are necessarily blind to agents’ mental states (see Himma, 2009 re. the “other minds problem”), and conclude that robots who elicit these practices (responses) are fitting targets of them. This insight does not allow us to say that robots are thereby moral agents or morally responsible, or that they are fitting targets of the full range of our practices, or that they can fulfill all the functions which we tend to ascribe to mature, neurotypical human beings, however. Unlike my proposed account, it does not reveal the kind of moral competence to which our practices are sensitive.

Danaher (2020) prescribes ethical behaviorism, according to which we ought to attribute moral status to a robot if it behaves in a way that we interpret as a feature of those to whom we already ascribe moral status. If the capacity for suffering is grounds for moral status and a robot appears to be suffering, then we ought to attribute moral status to the robot. Since we attribute moral status to human beings on the basis of mental states which we can only infer from their behavioral representations, we ought to do so, Danaher argues, with humanoid robots. I disagree with Danaher’s normative stance; however, ethical behaviorism is an approach which respects our epistemic limits. Even if there are mental states that provide the ultimate metaphysical grounds for our ethical principles, we can only know them by way of their behavioral representations (Danaher, 2020: 2028). This is reflected in our social practices and especially in our tendency to anthropomorphize other beings and entities.

These practices – even when they err on the side of caution toward the agent in question (better to treat someone/something well just in case it is sentient or conscious, etc.) – come with risks. For one, we risk expending our resources on those who cannot reciprocate them and for another, we are vulnerable to malicious deception, e.g., something that emulates pain could lure in and harm an unsuspecting good doer. There are other normative reasons (as pointed out in Darling, 2016 and Coeckelbergh, 2021) to avoid destructive behavior toward robots – human agent-centered reasons (relating to how our behavior affects our own character or moral worth) – but Danaher’s approach captures something descriptively significant about our practices; we judge others based on very limited and fallible inferences. The reason that supports doing so with non-humans is that it appears to be our only means of ascertaining the morally relevant information.

Mark Coeckelbergh’s earlier proposal (Coeckelbergh, 2009) of virtual agency and responsibility falls along similar lines. Coeckelbergh takes our existing social practices of ascribing these concepts to others as his theoretical starting point, observing that within human interactions, we ascribe agency and responsibility independently of “the real” (Coeckelbergh, 2009: 184). They are in this sense virtual concepts; we ascribe them to others on the basis of how we experience them and how they appear to us. Since we engage in these virtual ascriptions with humans and animals – often going so far as to attribute a will to the latter – we will (and do) and further, should engage likewise with robots. Since moral responsibility and agency are, as far as our means of assessing them goes, matters of appearance and performance, Coeckelbergh argues that our ascriptions of these concepts or features to robots ought also to be a matter of appearance and performance.

My proposed account follows what I take to be a related yet distinct approach. Rather than starting with a particular conception of moral agency as per the standard view, or investigating solely whether robots could fulfill the functions we ascribe to morally responsible agents, or as do Danaher and Coeckelbergh – taking our practices themselves as the basis for a prescriptive account of artificial moral agency – I utilize the Strawsonian methodology by taking our responsibility practices as a starting point and identifying the criteria at work in them, in order to determine the features of a morally responsible agent. Like Danaher and Coeckelbergh, I note that our practices are limited to behavioral assessments and as such, they do not sufficiently capture or reflect all morally relevant mental content.

However, I do not take our practices to settle the matter about moral agency which, like Peter Asaro (2006), I consider to be a scalar phenomenon – with moral autonomy on the upper end of the spectrum. I take them to set the standards for morally responsible agency, which I take to be lower than for moral autonomy. I hold that we ought to adopt further practices (and revise existing ones where possible^12^), in order to cultivate moral autonomy – something I do not see as a realistic (or desirable^13^) goal for robot design. Although certain robots are in principle capable of passing the performance-based test for morally responsible agency, I do not argue that this is a sufficient basis for the rights and privileges that other moral community members may be owed in virtue of other features such as sentience or personhood. So although this approach provides an answer to whether robots pass the Strawsonian requirements for moral community membership, it does not investigate whether this is in fact a desirable outcome. I will, however, put forward a conception of morality (in Section 5) as well as practical reasons (throughout), which offer practice-independent support for behavioral moral agency as morally responsible agency and as a potentially sufficient basis for moral community membership.

Before moving on, however, it is important to situate my proposal with respect to the other recent accounts of artificial moral responsibility previously mentioned (List, 2021; Tigard, 2021). Christian List argues that certain artificial intelligent systems, like certain group agents (e.g., corporations), who meet the following conditions on responsible agency are morally responsible: 1) moral agency, 2) knowledge, and 3) control. Respectively thus, the entity has to be capable of 1) making normative judgments about its choices and responding correctly to those judgments, 2) obtaining information relevant to this normative assessment, and 3) of being in sufficient control to choose between its options (List, 2021: 16). Which entities meet these conditions is ultimately an empirical matter, List concedes, but he does not see any *a priori* reason to deny moral responsibility to those entities which could be shown to meet them. List considers that the moral agency condition can be met in the form of compliance departments and ethical committees (in the case of corporate agents), rendering it a condition which can be plausibly met by other types of artificial agents as well. This notwithstanding, List is careful to point out that currently feasible artificial agents lack what he takes to be the requisite feature for intrinsic moral significance (phenomenal consciousness) and are thus excluded from the full range of protections and privileges we grant those who have it.

Daniel Tigard (2021) also argues in favor of artificial moral responsibility, but rather than starting from a set of necessary conditions for responsible agency and seeing whether artificial agents can meet them, he takes an ecumenical Strawsonian account of moral responsibility (Shoemaker, 2015) which can accommodate a plurality of agents, and argues that it can be extended to accommodate certain artificial agents as well. According to this account, there are different faces of responsibility – attributability, accountability, and answerability – each of which tracks different agential features – character, regard for others, and evaluative judgments, respectively. Hence, an agent who lacks the feature required for accountability-responsibility (regard for others) might nonetheless be responsible in an attributability or an answerability sense. Tigard suggests that artificial agents with one or more responsibility-relevant feature can thereby qualify as responsible, in that respect.

Diverging from List, who identifies *a priori* what features are required for moral responsibility and then makes a case that artificial agents can meet them, both Tigard and I employ the Strawsonian approach to responsible agency as being a matter of what our practices track. On my analysis, our practices track our capacity to comply with normative demands and, what is more, they cultivate this capacity. Shoemaker’s account admittedly offers a richer, more nuanced analysis. However, although his analysis reflects a wider range of psychological and moral commitments, it overestimates the reflective sensitivity of our practices and neglects their regulative power. On my view, our responsibility practices can neither sufficiently reflect nor direct agents’ mental content and consequently, they reflect and cultivate only behavioral moral agency. Finally, on the view that Tigard adopts, agents who have particular responsibility deficits – agents on the margins (as Shoemaker puts it) – would not necessarily pass a threshold for responsible agency (should there be one) and would thus have restricted agential status within the moral community. By contrast, my analysis of our practices entails that morally performing agents meet that threshold.

## 3 The Regulative Nature of Moral Responsibility Practices

The Strawsonian approach takes our practices to reflect responsible agency. Like the moral responsibility consequentialists (Schlick, 1939; Smart, 1961; Dennett, 2015) and instrumentalists (e.g., Vargas, 2013; McGeer, 2019; Jefferson, 2019) and Hume and Hobbes before them,^14^ Strawson (2008) acknowledged the regulative power and social utility of our responsibility practices. He simply contended, contra “the optimist” (i.e., the consequentialist), that it would be wrong to account for our practices solely in terms of their effects, as that would undermine their expressive function and their roots in our beliefs – not about regulation – but about desert, responsibility and justice. Additionally, there is the communicative dimension of our practices (Watson, 2004; Darwall, 2006; Shoemaker, 2007; McKenna, 2012), according to which they constitute a form of moral address – communicating moral expectations and demands and sustaining interpersonal relationships. However, as the instrumentalists argue (McGeer, 2019; Jefferson, 2019), the regulative effects of these practices are no mere side-effects; our responsiveness to them is constitutive of responsible agency. Furthermore, these practices are necessary for the development and maintenance of responsible agency (Dennett, 2015; McGeer, 2019).

Though I do not deny their expressivist and communicative functions, it is the instrumentalist focus on the role of our practices on moral development that I adopt here. However, whereas the instrumentalist views our practices as necessary and sufficient conditions of robustly responsible agency, I see them as merely necessary. I also claim that they work, in some ways, against the development of moral autonomy. They are necessary because they communicate the normative landscape (Sie, 2018; Sliwa, 2019), regulate behavior in ways that enable internal regulation and reasons-responsiveness, and forge a connection between morally relevant social feedback and behavior. They are insufficient because they cannot enhance moral reasons-responsiveness directly. They are sometimes counterproductive to autonomy because they regulate behavior via conditioning and may impede moral reasons-responsiveness. Briefly, the argument that our practices cannot directly enhance moral reasons-responsiveness goes as follows.^15^

A responsibility response like blame or resentment is surely involved in communicating the normative landscape to developing agents. We can assume, however, that mature wrongdoers, absent excuse, were aware of the relevant moral reason at the time of wrongdoing, in which case the response does not serve to communicate a new moral reason. Although I take it that our responsibility responses are indicative of wrongdoing (we feel resentment, e.g., toward someone who has behaved badly), I hold that they stand at some remove from moral reasons themselves. So with developing agents, resentment (e.g.,) may accompany the moral reason and with both developing and mature agents, resentment may communicate additional moral obligations to the wrongdoer which have been incurred by the wrongdoing – e.g., obligations to express remorse, apologize, reform, etc. However, responsibility responses (reactive attitudes) are not the moral reasons at stake in the wrongdoing and thus can only be paired with moral reasons.

Consider the case of breaking a promise – say to help a friend move, in favor of some selfish motive – say staying on at the sports bar to catch the end of the match. Suppose the motive behind the broken promise comes to light and the promisee resents his friend. The resentment communicates the promisee’s disappointment and places the wrongdoer in a position to take further action (expressing remorse, apologizing, promising to uphold promises in the future) should he wish to repair his relationship and moral status. Taking further action manifests regard for the promisee and though the wrongdoer displayed insufficient regard in the initial wrongdoing, I maintain that a general regard for others is insufficient for all our moral obligations (i.e., you can commit a moral wrong while manifesting regard for another’s well-being by e.g., lying to spare their feelings). Promise-breaking is wrong irrespective of whether the motive behind the wrongdoing comes to light or the promisee experiences resentment (or even whether a hypothetical agent experiences resentment). Provided thus that our responsibility responses are not themselves the moral reasons at stake in the wrongdoing, and can only accompany moral reasons (or present additional moral reasons), they do not enhance moral reasons-responsiveness directly. Indirect influence cannot guarantee concrete outcomes.

Instead, I suggest, more straightforwardly, that our responsibility responses directly influence only behavior. A behavior cultivation model has a clear evidentiary advantage over the moral reasons-responsiveness cultivation model since we cannot observe agents’ mental content directly. On the behavioral model, our practices influence behavior directly by pairing a non-moral reason – the sting or pleasure of the response (e.g., blame or resentment, praise or gratitude) – with the wrong- or right-doing.^16^ An agent need only be sensitive to the emotions and opinions of others in order to modify their behavior accordingly. In principle, the higher this sensitivity and the stronger the response, the greater the sting (in the case of blame or resentment) to the wrongdoer, and the stronger a reason to avoid future wrongdoing. In essence, therefore, our responsibility responses require sensitivity, not to moral reasons, but to the pleasure and pain of social approval and disapproval in order to be shaped by them. The very principle of behavioral conditioning is that the reinforced behavior remains after the reinforcing stimulus has been removed. In this respect, we are programming one another,^17^ via the moral responsibility practices, to behave according to rules and values rather than to act for the moral reason.

As a brief aside, this description of how our responsibility responses shape behavior may trigger skepticism on the part of the reader as to how non-sentient beings might be responsible. They would not, after all, have the constitution of a human responsible agent – sensitivities to pain and pleasure, approval and disapproval. Though this issue will be addressed in other parts of the paper, a brief clarification is in order. Human moral compliance requires these sensitivities (at least until a feedback independent knowledge of moral reasons and a sensitvity to those reasons arise); machine moral compliance does not. That is not to say that machines need not have “sensitivities” in terms of responsiveness to their programming, but this responsiveness need not resemble ours.

Well prior to being able to grasp the moral significance of our actions, we are made, in virtue of these sensitivities, to comply with moral norms. When we are very young, this process is undertaken by our parents and caretakers via the imposition of sanctions and rewards. “Habituation into virtue works because emotional rewards and sanctions gradually alter a person’s affective responses and motivational tendencies, in ways that can correct them” (Jacobson, 2005). Once (if) sufficiently habituated to right behavior, we develop an increasingly reasons-responsive disposition and the ability to regulate ourselves. Accordingly, mature agents are not taken to be fitting targets of behavioral management. By a certain level of maturity, educators and caretakers (should) attempt to provide deeper explanations about the moral significance of the actions upon which we impose sanctions and rewards. We hope that over time, children will be motivated by the right/wrong-making features of actions directly. We expect that adults follow laws and moral rules, not out of any fear of getting caught and sanctioned or out of a desire for praise and reward, but out of a deep and well-founded respect for the rightness of those laws and rules (when, of course, those laws and rules are right). We further hope that we will have the capacities to challenge and change those laws and rules which are unjust.

These hopes notwithstanding, by the very nature of behavioral conditioning, as stated, reinforced behavior remains after the reinforcing stimulus has been taken away. Once the connection between action and consequence has been forged, what reason motivates the action – whether the moral reason or the reason tied to the externally imposed (secondary) consequence – may be impossible to discern. On the view that behavior that corresponds with moral norms is moral (or virtuous), this is an unproblematic outcome (from a consequentialist perspective, at least). On the Kantian view, only morally autonomous action – action performed for the moral reason – has moral worth. Any action performed as a result of a law imposed externally (e.g., by means of a sanction) is morally heteronomous (Korsgaard, 1996: 22). However, from an epistemic standpoint, our appraisals of others are generally limited to observables and thus to behavior. We cannot observe reasons for action.

Our very development as moral agents is thus highly dependent, at least early on, on conditioning practices and our means for appraising moral agency, largely limited to appraising behavior. This is not to say that we don’t value acting for the relevant moral reason over the prudential one. Our theories of praise and blameworthiness make this distinction; it’s our responsibility practices that cannot sufficiently apply it. Furthermore, sanction and reward may well be deeply connected to moral reasons. As previously argued, however, what makes wrong actions wrong and right actions right stands at some remove from sanction and reward and from the reactive attitudes manifested by others. Finally, I suspect that many moral agents develop beyond mere behavioral moral agency. If they do, however, it is likely thanks to something other than what the responsibility system – based on sanction and reward as it is – can provide. Whatever this something consists in, it likely involves institutional support and material conditions with which not all are provided.

## 4 Mechanisms of Regulation and Their Limitations

In this section,^18^ I address specific features of our responsibility practices which are conducive to a behavioral species of moral agency. First, as conditioning practices, they shape and confine developing agents’, in particular, choices. For those who have experienced rewards for certain behaviors will likely be more attentive to these options than those who have not. Still, conditioning does not necessarily bypass the deliberative process. One may contend that anything short of physical coercion shouldn’t count as true coercion (Watson, 2004). But the reason for engaging in or avoiding behaviors which have been directly appraised, if the appraisal is effective, might easily become the pursuit or avoidance of these responses, not the moral reason. In fact, our responses may take our attention away from the moral reason, decreasing moral reasons-responsiveness. The fear of social embarrassment alone may easily outweigh a concern for the right reason for one who is not already sufficiently robustly moral reasons-sensitive, and any true wrongdoer is, by definition, insufficiently responsive to moral reasons.

Another agency-defining feature of our practices is their prioritization of behavior over reasons for action and the role this plays in promoting behavioral conformism. As Danaher and Coeckelbergh point out, this prioritization is due in part to our epistemic limitations. In general, we are blind to agents’ true motives. It’s not to say that we do not care about them and we can of course solicit them from agents post-factum. However, 1) this is generally done only in the case of wrongdoing; we tend not to solicit reasons for right actions, i.e., we tend to take for granted that good-doers have acted for the moral reason and not e.g., to impress their peers.^19^ 2) Such testimony is unreliable; we tend to provide post-hoc rationalizations of our behavior (Haidt, 2001), and 3) this is generally relevant only to the way we adjudicate punishment, not to the initial appraisal and response. In general, we attend more to apparent wrongdoing. There is a well-known prioritization of blame (over praise) in our practices (and theories). Moreover, due to 1) above, we often bestow praise upon actions which appear morally worthy even when they’re not (e.g., when someone is helpful because they care what by-standers think of them). Even when we don’t offer praise, though, by not-blaming these actions, we express approval nonetheless. We thereby promote behavioral conformism, reinforcing behavior which merely conforms with moral values – irrespective of an agent’s reasons for acting.

Third, behavioral conditioning via these practices can address only a very limited set of morally-salient behaviors. Insofar as we are wholly dependent on these practices to learn the normative landscape, they can thus provide only limited moral development. 1) Their scope is limited to the domain of past actions. We cannot directly influence behaviors which have not occurred. Of course, by letting others know how we will feel or react should they behave in a given way, we can influence their future behavior. a) This would likely be a weaker form of influence than direct, emotional responses and b) the domain of influence is limited to that which can be anticipated and articulated. This form of influence, though part of the inter-personal realm, is akin to the way our society manages our environments, placing limits and negative incentives on certain actions. By including moral reasons and principles of right action along with our responsibility responses, we can target a much wider range of moral behavior. However, provided that we are dependent for right action on these responses (something which is assumed by McGeer, 2019 in her scaffolding view of responsible agency), then our moral agency cultivation remains limited in scope. 2) Acts and expressions of moral condemnation and praise target behavioral outliers – behaviors which transgress or exceed our moral expectations and, of course, only those that are visible to us. On the other hand, not-blaming conformist behavior reinforces it.

Fourth, in order to legitimately hold others to account (e.g., via blame or punishment), we require strong degrees of confidence in their guilt. Although we probe an agent’s motivations more deeply in the case of wrongdoing, if we probe far enough below the surface of an agent’s history, upbringing, environment, motives, etc., such confidence is hard to come by. Consequently, we tend to base that confidence on seemingly obvious, clear-cut, superficial information about an agent (how the agent appears to us, our perception of their quality of will and motives of action) rather than the deeper but likely truer causal factors at play (see also Dennett, 2015). The result is a restricted set of criteria for our moral responsibility practices which, in turn, fosters a restricted (behavioral) species of agency.

## 5 The Case for Robot Responsibility and Community Membership

As previously stated, on my view (cf. Asaro, 2006), moral agency arises on a spectrum. At the high end of the spectrum is moral autonomy. Somewhere along the spectrum before moral autonomy, the point at which we reach a certain threshold of moral competence, we become morally responsible.^20^ I suggest that this competence is the capacity to reliably behave according to moral norms. Without this capacity, we are not morally responsible for our actions and are thereby excluded from the moral community. I argue that moral rule-abiding robots that have the capacity to uphold social role-specific normative expectations are thus morally responsible. According to the Strawsonian notion of moral community as a matter of moral responsibility, morally responsible agents are moral community members too.

Though I leave open the possibility that responsible agency and moral community ought to come apart, there are some normative reasons to keep responsible agency as a sufficient condition of community membership. 1) Responsible agents (agents who can reliably behave in accordance with norms) contribute to the realization of the ethical aim of social cooperation.^21^ 2) Demanding more than responsible agency is to demand something our practices cannot (and, in liberal societies, should not attempt to) regulate. Our social responsibility practices regulate behavior (presumably, for the sake of social cooperation). By definition, moral autonomy is not something that can be imposed externally on an agent; it requires that agents be motivated directly by moral reasons. Although there are surely necessary external conditions for the development of moral autonomy (e.g., the right upbringing, a scholarly study of the good,^22^ practices which draw our attention to the direct harms and benefits of our actions, thereby cultivating a concern for moral reasons directly rather than for sanctions and rewards), these conditions are not only not guaranteed, they offer no guaranteed outcomes. 3) More troubling, we cannot see or verify whether moral reasons are the motivating reasons. We are largely limited to evaluating and thus enforcing agents’ performance.

In support of 1), I offer P. F. Strawson's Strawson (2008: 5) basic conception of morality.^23^

“Now it is a condition of the existence of any social organization, any human community, that certain expectations on the part of its members should be pretty regularly fulfilled; that some duties, one might say, should be performed, some obligations acknowledged, some rules observed. We might begin by locating the sphere of morality here. It is the sphere of observation of rules, such that the observance of some such set of rules is the condition of the existence of society. This is a minimal interpretation of morality. It represents it as what might literally be called a kind of public convenience: of first importance as a condition of everything that matters, but only as a condition of everything that matters, not as something that matters in itself.”

According to Strawson, then, morality in its most basic terms^24^ – the observance of a certain set of rules which makes society possible – makes possible the higher human goods. A moral agent is thus, first and foremost, an agent who follows and whom we expect to follow these rules.^25^ Whether a moral agent could or should pursue moral autonomy is irrelevant to their status as a moral agent. Strawson (2008) argues that our moral responsibility responses are reactions to the fulfilling, exceeding, or transgressing of our normative expectations about how others will behave. Moral rule-abiding robots can meet our basic normative expectations and thus support social cooperation.

For humans, meeting these expectations – acting in accordance with moral norms – is no straightforward matter. With robots, again assuming the formalizability and programmability of moral norms, such behavioral compliance is a product of design. This is at odds with a conception of morality as tied to freedom and yet, as previously argued, we are attempting, via the conditioning of the responsibility practices, to program human beings to comply with moral norms too. However, this form of programming can be viewed as a kind of “weak programming” that does not preclude an agent’s capacity to alter course. Matheson (2012) argues that sufficiently complex robots can be viewed as weakly programmed as well and so, insofar as humans are weakly programmed and yet morally responsible, so are such robots. As Susan Wolf (1980) has shown, being determined to act morally – as in the case of someone who is incapable of cruelty – is not at odds with moral responsibility; an agent determined in this way is still praiseworthy for their virtuous actions.^26^ Finally, to repeat an earlier point, acting against a moral reason and in favor of a selfish impulse is indicative of an agent’s lack of moral autonomy.^27^ A morally autonomous agent is ultimately responsive to the relevant moral reason. Hence, although I don’t consider the robots under consideration in this paper to be morally autonomous, since they are not able to give themselves the moral law (Korsgaard, 1996)^28^, their status as programmed entities does not preclude their morally responsible status.

Mature, neurotypical adults are taken to be morally responsible even when they don’t behave morally. Moral rule-abiding robots, however, I claim are morally responsible because they have the capacity to reliably behave according to moral norms. As stated previously, it is precisely this capacity that qualifies human agents as responsible agents (see also Dennett, 2015). When a responsible agent transgresses a moral norm, we blame them. However, what renders them liable to blame is their status as a responsible agent, and what gives them this status – machine or flesh and blood – is the capacity to reliably behave according to moral norms. I hold that adults who consistently transgress moral norms, despite being treated as morally responsible, lack this capacity.

At this point, the elephant in the room should be addressed with more than a footnote: whether robots might be capable of acting on moral norms. A significant source of skepticism regarding whether they can rests on the claim that moral agency is a matter of acting for the right reasons which, in turn, requires consciousness (Purves et al., 2015) or the ability to e.g., perceive certain facts as moral reasons (Talbot et al., 2017).^29^ Since robots lack these capacities, they lack the relevant capacities for moral agency. On my account, however, responsible agency is a matter of behavior – not mental content. Hence the moral competence of concern to my account is one of performance.

But the elephant remains in the room. Can robots comply with moral norms? And this becomes a matter of whether moral norms can be codified and programmed and then autonomously applied in relevant situations, or whether a design architecture can accommodate learning moral norms from the data and then applying them. Unfortunately, these questions are beyond my expertise to answer; fortunately, they are being addressed.^30^ Moreover, there are reasons for optimism on this front, as some autonomous machines (e.g., self-driving cars) are already able to operate relatively reliably in morally and socially significant ways and contexts. They could thus be said to have the moral competence I have argued is relevant to responsible agency. Joanna Bryson’s normative argument against the creation of artificial moral agents (see e.g., Bryson, 2018) offers indirect but significant support for the belief that we have or will have the capacity make machines which could behave according to moral norms.

Finally, it is possible that many mature, reasons-responsive agents whom we deem morally responsible are not sufficiently internally regulated or responsive to specifically moral reasons. Our society accordingly manages their behavior by a slightly less visible set of strings – by establishing consequences (largely sanctions) to be imposed by legal and social institutions and by relationship partners (in the form of the negative reactive attitudes if nothing else). Without a reliable means to secure robust responsiveness to moral reasons, it is necessary (and likely more expedient) to rely on our natural aversions to sanction and desires for reward in order to ensure societal cooperation. Because the moral responsibility practices are regulative and they set, enforce, and reinforce the standards for moral agency and thus moral community membership, they largely both reflect and determine society’s level of moral development. Whether this is ultimately desirable is another matter. The point is that the standards at work in our social practices are such that moral rule-abiders qualify as moral community members and what’s more, enable social cooperation.

## 6 Shortcomings and Possible Solutions

Even should one accept the claim that moral responsibility requires only behavioral moral agency and that some robots can thus be morally responsible, there are moral responsibility functions in terms of accountability which cannot be satisfied by robots. There are of course instances of primitive artifacts (like sex dolls, as noted in Nyholm et al., 2019), not to mention sophisticated androids, which can and will inspire what a human counterpart takes to be a reactive attitude like love. This may not be “genuine love,” but even assuming that it is, it’s not clear that such a robot could inspire our full range of reactive attitudes. Even if a robot were causally responsible for a mass killing, it’s far from certain that we would see any purpose in holding it accountable via blame or punishment. We would, where possible, hold the human moral agents behind the robot morally and criminally responsible. How can we call responsible an agent whom we would not blame, especially if our criteria for responsibility are tied to our practices of holding responsible?

I can offer two answers. 1) As I have argued, having the capacity to reliably behave according to moral norms qualifies one as morally responsible. This claim rests on a distinction made by Angela Smith (Smith, 2007) between the conditions for responsible (blameworthy) agency and the conditions for active blame. The ensuing “gap between conditions of culpability and appropriate blaming, Smith argues, shows that conditions of being responsible cannot be reduced to conditions of appropriate active blaming” (Russell, 2011: 211-212). Hence, robot responsibility is not obviously precluded by the possibility that it might never be appropriate to actively blame them.

2) Our practices are imbued with persistent incompatibilist (libertarian) intuitions. We mistakenly believe that a wrongdoer had the power to do otherwise. Interestingly, responding from this belief to the wrongdoer may be essential for securing forward-looking benefits; i.e., we may have greater success in preventing future wrongdoing when we authentically resent someone for it. Authentic resentment may depend on believing that a wrongdoer ought to have done otherwise. Responding as if the agent really deserves blame or sanction may not only be our only option, psychologically speaking, it may also be the most optimal means of shaping behavior. This said, the very concept of just deserts is the issue at stake in the moral responsibility debate. Would it be fair to blame or punish someone who lacks sufficient control over their character or actions?

The traditional compatibilist says that although we lack ultimate control, we have enough control (or the right kind of control, e.g., guidance control; see Fischer and Ravizza, 2000) to deserve being blamed for our wrongdoings. The instrumentalists, however, have other resources to justify our practices of holding responsible. In conclusion, by rejecting the traditional justification for desert-entailing moral responsibility, we are free to embrace the forward-looking dimension of our practices and hence, the fact that we may not see a backward-looking purpose in holding robots accountable, is not fatal to their moral responsibility. We must thus also consider, which I will do shortly, to what extent robots can be morally responsible in the forward-looking sense.

However, there are other functions served by holding others to account which the above answers do not address. They relate to the inadequate “psychological machinery” (Babushkina, 2020) possessed by foreseeable robots. One such function concerns our primal retaliatory urge, something we share with some primates which, when acted upon, has certain proven physiological benefits for the avenger. This gives rise to what John Danaher (2016) refers to as the “retribution gap.”^31^ However, revenge practices are also at the root of vicious cycles of aggression and destruction (see Waller, 2012; Waller, 2015). In civilized society, we deter individual acts of revenge and adopt a collective, institutional approach which, though less satisfying to the individual, may nonetheless be characterized as serving a retributive aim. Despite the significant psychological relevance to our practices, as a morally suspect dimension of them (see e.g., Caruso, 2021), I will dismiss this worry as pertains to robots. The issue of adequate psychological machinery is bigger than retributivism, however. Without it, as pointed out by Dina Babushkina (2020), meaningful accountability practices are impossible. Not only can robots not feel the sting of condemnation or punishment or be brought to suffer by them, they cannot feel guilty or, in turn, be forgiven. Blaming robots would thus create a kind of “blame vacuum” and per Danaher (2016) and Babushkina (2020), lead to moral scapegoating.

On the communicative conception, blame is a form of moral address and concerns the blamer and the blamee. Both parties must meet the criteria required for their respective roles. Could a robot meet the criteria for either role? I will focus here on the role of blamee, for it gets to the heart of the concern in the machine moral responsibility debate. According to Coleen Macnamara (2015: 212), eligibility for this role “requires the capacities necessary to give uptake to the distinctive form of communication that reactive attitudes constitute. Uptake of the reactive attitudes amounts to feeling guilt and expressing it via amends, and to respond to blame in this way requires moral competence.” Although triggered by a past action, moral address presents forward-looking reasons – apologizing, the making of amends, offering compensation, promising reformation. It is conceivable that robots could fulfill these obligations, at least performatively, but the above objection – when it comes to the psychological dimension of blame – still holds.

To this objection, I offer the following. 1) On the communicative conception, blame is a two-way street and requires certain symmetrical capacities as relates to communication. Part of the communication is strictly emotional – and the blamer would likely not be psychologically satisfied or able to forgive based on what they take to be a mere performance of guilt or sorrow. Mark Coeckelbergh might respond that robot designers ought to aim for an authentic performance capability and, if successful, it may in fact satisfy the psychological dimension of blame (resolving the “blame vacuum”). If it is not successful, then we face the same problem we presently face with many existing moral community members from whom, when they make moral mistakes, we cannot get the satisfaction or resolution we want from holding to account. This group may include those who have fallen on hard times, those struggling with addiction, mental disorder, poverty, social isolation, poor formative circumstances, toxic social environments, etc. With them, we must establish alternative ways of managing our psychological needs – ways which could then be extended to robots to some degree.

However, if we – as a society – have not reached a legitimate consensus about what societal functions robots ought to fulfill and which robots ought to fulfill them – in light of ultimately transparent and relevant risk-benefit analyses – the blame vacuum is likely to create significant societal problems which I cannot dismiss here. Provided a legitimate consensus, dealing with negative outcomes may be psychologically less challenging than dealing with negative outcomes – even those resulting from strictly human actions – over which we’ve exercised no agency. 2) Scapegoating is part of the more general responsibility gap problem present in complex technological chains (Matthias, 2004), not just in the context of artificial moral agents. This gap extends well beyond the robot question, through to collective agents and, as I’ve suggested, to individuals within the human moral community as well.

Where to draw the line for individual human responsibility is no easy task, whether due to determinism or indeterminism in our causal histories. If responsible agency requires a particular psychosocial constitution and careful conditioning practices, which in turn must be tied to the right moral norms, it is likely that many among us are unable to develop responsible agency. For these agents (as well as the abovementioned agents), we need “alternative accountability structures” – social institutions which take responsibility for those who cannot – both the wronged and the wrongdoers.^32^ These structures could help equip wrongdoers (or otherwise stand in on their behalf) to fulfill the obligations which their wrongdoing has incurred, in addition to providing them with resources to develop moral competence. These structures should provide recourse to those who have been wronged and who cannot obtain what holding to account should make possible. Such structures could be called upon in the case of robots.

On my account of moral agency, moral autonomy is the level of agency required to be responsible in the deepest sense of the term – to be substantively in control of one’s actions and characters^33^ – and this level of agency requires practices and conditions on top of our responsibility practices. In this light, only a portion of our moral community is substantively responsible. The majority of the rest of our community has the moral competence to conform to the rules and thus to respond adequately to our practices; the minority does not. The line between these latter two groups, however, is likely very blurry. By minimizing or eradicating traditional desert-based practices and maximizing the forward-looking ones, we reduce the risks of mistaking where this line, if it exists at all, is.^34^

I now return briefly to the forward-looking goals of reformation and restoration independently of any alternative accountability structures. First, robots could be equipped with a primitive reinforcement learning architecture whereby our negative reactions would serve to prevent their negative behaviors in the future (Gogoshin, 2020; Tigard, 2021; see Wallach and Allen, 2009 for an example). Reforming robots directly via the moral responsibility responses is thus conceivable. Moreover, on an instrumentalist account of responsible agency as a matter of susceptibility to our responsibility practices (see Schlick, 1939 for an early version; see Jefferson, 2019 and McGeer, 2019 for more nuanced contemporary versions), robots who could be designed to adequately respond to our practices^35^ could thus qualify as fully responsible agents, though not in a way that would satisfy all our folk intuitions and practices. An instrumentalist, however, is well positioned to argue for a revision of our practices. Finally, it is also conceivable that robots may be conscripted to do more extensive (and potentially hazardous) acts of restoration than humans. Hence their forward-looking responsibility may be, in some respects, greater than ours.

The complexity of this discussion, due foremost to the ambivalence which envelops moral responsibility independently of robots, provides an especially weighty reason to reach societal consensus about what roles we want robots to fulfill and what the risk-benefit analysis of having them in these roles amounts to. This would allow us to put the necessary responsibility structures in place such that we can nip at least a bulk of the potential problems in the bud.

## 7 Conclusion

In this paper, I have argued that the level of moral agency required for moral community membership, insofar as that membership is a matter of responsible agency, is behavioral moral agency. This conclusion is a result of an analysis of the ways our moral responsibility practices function – both in terms of reflecting and fostering moral agency. Given 1) a methodology which takes our practices as evidence of responsibility and the fact that these practices largely address behavior, 2) a conception of morality as a set of rules which enable social cooperation, and 3) the Strawsonian picture of moral community as being a matter of responsible agency, the view that moral rule-abiding robots are responsible and thus moral community members, becomes plausible. Our commitment to moral autonomy necessitates at least two overlapping but distinct conceptions of moral agency. Traditionally, morally responsible agency has been taken to be full-blown moral agency requiring substantive freedom or control, but if our practices are the theoretical starting point, on my analysis of them, this view is incorrect.

I have conceded that robots are unlikely to satisfy all our accountability-responsibility demands. Accordingly, it is vital that we reach the societal consensus described previously. I further proposed what I would propose for the many human moral community members who also lack some degree of accountability-responsibility: alternative accountability structures. Finally, I suggest that we devote resources to the cultivation of human moral autonomy while keeping the bar for moral community membership at responsible agency (as I have defined it herein). This meshes better with our existing moral community, though it also accommodates morally performing agents of any make or model. If this is objectionable, we ought to redefine moral community membership in other terms than morally responsible agency or morally responsible agency in other terms than our responsibility practices.

## Data Availability

The original contributions presented in the study are included in the article/Supplementary Material, further inquiries can be directed to the corresponding author.

## Footnotes

1 By moral responsibility practices, I mean moral approbation and disapprobation, praise, blame, sanction and reward and the reactive attitudes (e.g., resentment, indignation, love, gratitude, etc.).

2 Though there may be other relevant artificially intelligent systems, I limit my argument to robots that meet Sullins’ definition of autonomous robots (Sullins, 2011: 154). “Autonomous” here refers to the roboticist or engineering sense in which Sullins uses it (see also Arkin, 2009).

3 A matter which, admittedly, remains far from settled; see Sharkey (2020) for a sobering overview of the current debate. Ron Arkin (2009) makes the most confident case for robot ethicality; see also Nadeau (2006). I address this further toward the end of Section 5.

4 According to Strawson (2008: 17), moral responsibility is a precondition of being “a term of moral relationships” and a moral community member. Zimmerman (2016: 251) states that Strawson takes all three concepts as synonymous. Per Darwall (2006: 17), moral responsibility is being subject to the moral reactive attitudes, which “presuppose the authority to demand and hold one another responsible for compliance with moral obligations (which just are the standards to which we can warrantedly hold each other as members of the moral community).”

5 Among current technologies, self-driving vehicles come quite close to the robots I have in mind. They operate rather reliably in high stakes settings. I envision care robots as an imminent example. However, it is not clear whether there are any extant robots that meet all the relevant moral demands of morally impactful social roles or, for that matter, how wide the social context they are capable of performing reliably in should be in order to qualify as moral community members. High stakes social institutions require specialized skills and security clearance, from financial institutions, to legal (courtrooms, prisons, government offices), military, medical, educational, safety (e.g., land/air/sea traffic control), etc. institutions. As a result, most humans have highly limited access to society, but this does not preclude their moral community membership. Hence, what is to be understood by moral community is the community of responsible agents. The fact that moral rule-abiding robots could qualify (under my proposed view) as responsible agents provides an impetus to investigate the concept of moral community carefully and to make prescriptive claims which uphold our ultimate moral values. The present proposal does not perform this task, though it will present (in Section 5) some normative reasons why its conception of responsible agency might be a sufficient condition for community membership.

6 On the proposed view, responsible agents need not be moral patients. However, we might wish to make moral patiency a requirement for moral community membership.

7 See Gogoshin (2020) for a condensed version of the stronger argument—that moral rule-abiding robots are ideal moral agents per the moral responsibility system.

8 I subsequently refer to this conception as “robust moral responsibility” or “substantive responsibility.” I hold that it requires, *inter alia*, robust moral reasons-responsiveness, i.e., the ability to recognize and respond to moral considerations in a wide range of circumstances. To be substantively or robustly morally responsible is to be largely morally autonomous: governed/motivated by the moral reason directly. Compare also the Aristotelian ideal of the virtuous person.

9 See Champagne and Tonkens, 2015 and Himma, 2009; they argue that consciousness is only needed for moral responsibility (Behdadi and Munthe, 2020).

10 With some exceptions; see Behdadi and Munthe (2020: 199–200).

11 This seems right headed, for even if a clear determination is made that foreseeable robots cannot meet the criteria for moral agency that we take human beings to meet, or that robots cannot fulfill certain normative expectations, we are still faced with this question.

12 By setting strong limits on the degree of punishment, e.g., we administer (eliminating retributivism altogether), attending more, in the ways we can, to agents’ reasons, etc.

13 I hold that robots are desirable only as non-autonomous moral agents, subject to human moral demands since, if morality is a matter of a species’ flourishing, as a distinct species, autonomous robots would pursue their own flourishing. Their flourishing may be at odds with ours.

14 Following thinkers like Thomas Hobbes, Hume points out that rewards and punishments serve to cause people to act in some ways and not in others, which is clearly a matter of considerable social utility (T 2.3.2.5/410; EU 8.2897–98)” (Russell, 2021).

15 See Gogoshin (2021a) for an elaboration of this argument and the argument in favor of the behavioral model.

16 Along with Joel Feinberg (1970), I hold that expressions of blame are punishing. I further hold that expressions of praise are rewarding.

17 I address programming in Sections 4 and 5.

18 See also Gogoshin (2020).

19 Though here I make an empirical claim, I take it to be fairly uncontroversial.

20 As a reminder to the reader, by “moral autonomy,” I mean governed by (motivated by) the moral reason directly. A morally autonomous agent possesses the capacity to consistently act *for* (not merely in accordance with) the moral reason. This notion is compatible with the Aristotelian ideal of the virtuous person.

21 I realize that more than behavioral moral agency is necessary for moral progress, for which moral autonomy is necessary (Gogoshin, 2021b).

22 Following Aristotle in Book II of the *Nicomachean Ethics* (Aristotle and Crisp, 2014).

23 Thanks to a referee for pointing out two important sources of support for this conception: 1) the morality-as-cooperation view of anthropologist Oliver Scott Curry (Curry et al., 2019) and 2) Joanna Bryson's (Bryson, 2018) view of ethics as being society’s means of structuring and maintaining itself, and according to which what is moral is what is socially beneficial.

24 He acknowledges the inadequacy of this minimal conception of morality, but sees “considerable merit” in it as well.

25 See also Gogoshin (2020).

26 In Dennett’s words (Dennett, 2015: 227), “For Kant […] we are only *really* responsible for the right things we do.” Wolf provides the contemporary take on it. Like Kant, she does not hold that we are blameworthy for morally wrong actions (though she finds a way to preserve blaming bad behavior). On my view, there is no such asymmetry; however, I do not endorse desert-entailing responsibility. Hence, praise/blameworthy take on a different ring when I use them; i.e., they could stand in for morally right/morally wrong. They could also, taking an instrumentalist or consequentialist rationale, simply denote whether praising/blaming someone can (1) promote their reformation – whether, i.e., they have the right kind of constitution (sensitivities of the sort I have described) to be held morally responsible (Schlick, 1939; Jefferson, 2019) – or (2) be socially beneficial (Dennett 2015; Smart 1961).

27 This idea, as I understand it, is behind Nadeau's claim (Nadeau, 2006) claim that only androids could be truly moral.

28 “When you are motivated autonomously, you act on a law that you give to yourself; when you act heteronomously, the law is imposed on you by means of a sanction” (Korsgaard, 1996: 22).

29 Thanks to the referee who pointed out the need for a clarification here and recommended these references.

30 See Powers (2006) for a “Kantian machine.” See Arkin et al. (2012) for a concrete proposal for moral decision-making in autonomous systems. See Anderson and Anderson (2015) for a principle-based healthcare agent. See Malle and Scheutz (2014) for an environment/feedback moral learning architecture proposal.

31 Thanks to a referee for providing this reference.

32 This is similar to solutions proposed in response to the responsibility gap (see Behdadi and Munthe, 2020 for a summary). However, I see a responsibility gap even at the level of the human individual. Becoming a responsible individual is itself beyond the control of the individual and sometimes, due to factors beyond society’s control as well (e.g., natural misfortunes). Individual responsibility gaps thus abound and society must take responsibility for and within these gaps.

33 What Hans Jonas (2007) refers to as “substantive responsibility.” Compare also Bruce Waller's (Waller, 2012) “take-charge responsibility.”

34 See Gregg Caruso’s proposal (Caruso, 2021) for a strictly forward-looking, non-retributivist approach to responsibility and legal justice. He argues that, in the absence of free will, desert-entailing responsibility ought to be rejected. This approach resolves, at least normatively, the particular responsibility (retribution) gap noted in Danaher (2016).

35 There are many unsettled issues here: what counts as an adequate response, whether the end goal is behavior or full-blown (moral reasons-responsive) moral agency (which it is for McGeer, 2019). But this is a promising path to follow nonetheless for both roboticists and philosophers.
